# Impacts of Heat Stress on Rabbit Immune Function, Endocrine, Blood Biochemical Changes, Antioxidant Capacity and Production Performance, and the Potential Mitigation Strategies of Nutritional Intervention

**DOI:** 10.3389/fvets.2022.906084

**Published:** 2022-05-26

**Authors:** Zi-Long Liang, Fan Chen, Sungkwon Park, Balamuralikrishnan Balasubramanian, Wen-Chao Liu

**Affiliations:** ^1^Department of Animal Science, College of Coastal Agricultural Sciences, Guangdong Ocean University, Zhanjiang, China; ^2^Department of Food Science and Biotechnology, College of Life Science, Sejong University, Seoul, South Korea

**Keywords:** rabbit, heat stress, production, mitigation strategy, nutritional intervention, immune function, redox status

## Abstract

Heat stress has become a widespread concern in the world, which is one of the major environmental stressors and causes substantial economic loss in the rabbit industry. Heat stress leads to multiple damages to the health of rabbits, such as organ damage, oxidative stress, disordered endocrine regulation, suppressed immune function and reproductive disorders, ultimately, induces the decreased production performance and increased mortality. Nutritional approaches, including feeding strategies, adjusting feed formula, and supplementing vitamins, minerals, electrolytes, Chinese herbal medicines, and functional active substances to the feed, were reported to mitigate the detrimental effects of heat stress in rabbits. Therefore, elucidating the damage of heat stress to rabbits; proper management and nutritional approaches should be considered to solve the heat stress issue in rabbits. This review highlights the scientific evidence regarding the effects of heat stress on rabbit's immune function, endocrine, blood biochemical changes, antioxidant capacity and production performance, and the potential mitigation strategies of nutritional intervention to alleviate heat stress in rabbits; which could contribute to develop nutritional strategies in relieving heat stress of rabbits.

## Introduction

During recent years, the rabbit meat production is growing in China and European countries to meet the increasing demands of diverse meat product, which has become a highly specialized industry (1). There are about 1.4 million tons of rabbit meat produced worldwide each year, of which China is the largest producer, and the Europe is the second largest producing region (2). Rabbit meat is considered to have good sensory properties, it is tender, lean and flavored (3). Rabbit meat contains less fat than other meat (e.g., pork and chicken) and rich in protein, unsaturated fatty acids, conjugated linoleic acid and minerals, which are easy for humans to digest. Also, the rabbit meat contains a lot of selenium and antioxidant vitamins, and rich in polyamine (4). Dietary polyamines can heal the wound in intestinal mucosa growth, maturation and regeneration (5). Considering these facts, many researchers and farmers have focused on the productive performance and carcass yield of rabbit meat during the last decades (3). The genetically improved rabbits in recent years have higher metabolic rates and production performances, making them sensitive to environmental stress, such as high temperature, transportation and changes in feed composition and intensive farming (6). These stressors affect the health and production performance of rabbits, and the negative impact of high temperature on production is prominent due to the thick villi and lack of sweat glands in rabbits; facing with the high ambient temperature, rabbits stretch out to lose heat by radiation and convection and raise their ear temperature, stretch the ear pinnae and spread them far from the body to expose the surface to the surroundings (7, 8).

**Figure 1:**
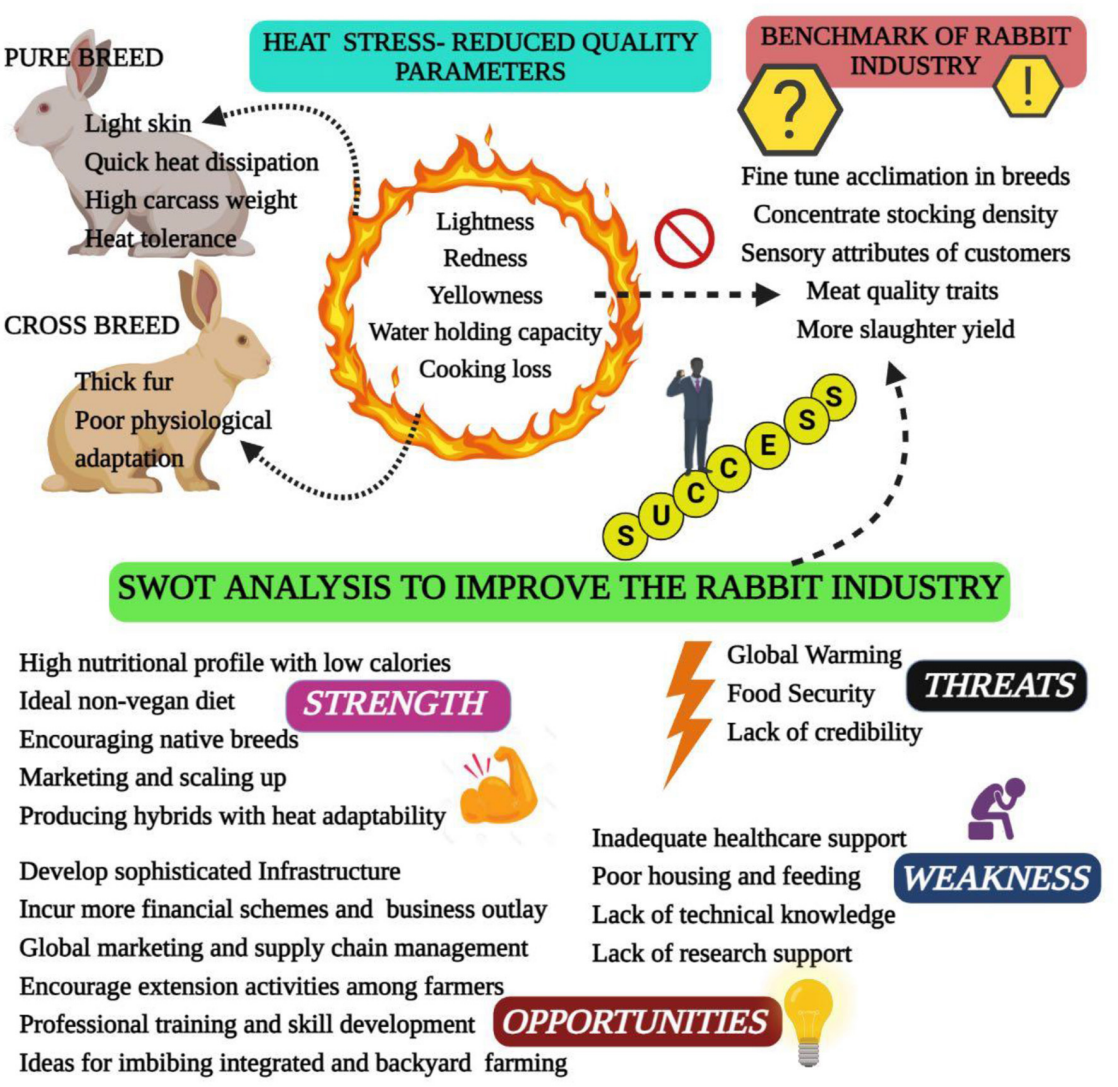
Challenges and opportunities for the rabbit industry in the context of global warming-induced heat stress.

Heat stress is a condition where rabbits are unable to maintain a balance between heat production and emission. High ambient temperature in summer is easy to cause heat stress in rabbits, which brings a series of adverse effects to rabbit production (6). Heat stress results from the interaction of different factors, such as high temperature, humidity, radiant heat and air speed. Of these, high ambient temperature plays a major role in leading to heat stress (9). The normal body temperature of rabbit ranges from 38.5 to 39.5°C, and the individual difference ranges from 0.5 to 1.2°C. The optimal temperature range of rabbits is 15–25°C, and the optimal humidity is 55–65%, heat stress occurs when the ambient temperature is higher than 30°C, when the temperature is higher than 35°C, rabbits cannot regulate body temperature, resulting in heat failure (7, 10). As known, heat stress has multiple unfavorable impacts on rabbit health and production performance, it has been suggested that heat stress causes 20–25% reduction in daily weight gain, 8–15% decrease in feed conversion ratio and 9–12% increase in mortality rate; and the reproductive performance decreased by 6–10%, as well as negatively influences the meat quality and carcass traits (6, 11, 12). Thus, heat stress causes great challenge for rabbit industry, especially with global warming (Figure 1). The potential mitigation strategies have been studied in the past, and the nutritional intervention proven to be effective mitigation approach (11). It is important to summarize these findings for rabbit researchers and industry. Therefore, this review focuses on the effect of heat stress on rabbit production and the potential mitigation strategies of nutritional intervention in rabbits.

## Impacts of Heat Stress on Rabbits

### Impacts on Immune Function and Endocrine

The immune dysfunction in rabbits caused by heat stress through the regulation of brain, sympathetic nerve and adrenal cortical hormone (11). The sympathetic adrenal medulla (SAM) axis is activated, which regulates the homeostasis in the early period of heat stress in rabbits, increased ambient temperature is perceived by the sympathetic nerves, transmitting the impulse to the adrenal medulla. The adrenal medulla increases the secretion of catecholamines, releasing glucose in the blood, depleting liver glycogen, reducing muscle glycogen, increasing respiration rate, vasodilating the peripheral blood vessels and increasing neural sensitivity to cope with the stress (13). When rabbits are exposed to a higher environmental temperature than their thermoneutral zone, the hypothalamic-pituitary-adrenal (HPA) axis is activated. The synthesis and secretion of hypothalamic adrenocorticotropic hormone releasing hormone (CRH) are increased significantly; CRH is used to act on the anterior pituitary to evoke the release of adrenocorticotropic hormone (ACTH) and it acts on the adrenal gland to promote the synthesis and secretion of glucocorticoid (14). Glucocorticoid provides the function of anti-immune response (15). The increased glucocorticoid inhibits cellular and humoral immunities, the protein synthesis in the lymphoid tissue and immune organs can be decreased, which eventually leads to a significant decline in overall immune function, and the weight of the thymus and spleen also decreases in heat stress treatment (16). Thus, heat stress reduces the immune function and causes rabbits are vulnerable to pathogens, bringing serious losses to rabbit production (17–19).

Under high ambient temperature, the synthesis of hypothalamic thyroid stimulating hormone (TSH) is highly reduced, resulting in a decrease of TSH in the anterior lobe of the pituitary gland and a reduction of thyroid hormone in rabbits; the decreased thyroid hormone could lower the metabolic rate and heat production of rabbits (20). In the early stage of heat stress, the body of rabbits accelerates the oxidation rate and peripheral circulation to resist the heat stress, and the levels of triiodothyronine (T3) and thyroxine (T4) were increased significantly, thus increasing the body's heat dissipation (20). With the extension of heat stress, the thyroid hormone level is gradually decreased (21). Reduced thyroid hormone affects the synthesis of protein (e.g., total blood protein, albumin and globulin) and causes the metabolic disorders of carbohydrates, fat and minerals in rabbits (22, 23).

### Impacts on Blood Biochemical Indexes

The blood biochemical index plays a crucial role in reflecting the metabolic changes and organ damage in rabbits under heat stress situation. The concentrations of total protein, blood glucose and triglyceride are decreased, whereas cholesterol concentrations are markedly increased during heat stress in rabbits (16, 24). These results can be attributed to the increased glucocorticoid secretion, which promotes gluconeogenesis process (25–27). In a previous study, immune cell proliferation and immunoglobulins synthesis were reduced, indicating the negative effect of heat stress on immune cell proliferation and differentiation (28). Yang et al. (29) showed that the differentially expressed genes in immune cells was involved in the cellular stress response, apoptosis, oxidative stress and glucose metabolism during heat stress. The concentrations of creatine phosphokinase (CPK), lactic dehydrogenase (LDH) and alkaline phosphatase (ALP) were decreased, and the glutamic pyruvic transaminase (GPT) and aspartate aminotransferase (AST) were increased in rabbits exposed to heat stress (30). The changes of these enzymes show significant liver damage and inflammation by heat stress in rabbits. In addition, heat stress causes alterations in minerals of rabbit plasma. Due to the increased ACTH by endocrine regulation, it promotes the glomeruli to retain sodium and excrete potassium so that the blood potassium level is reduced to maintain body fluid balance. Retaining sodium (Na) and removing potassium (K) increase the concentration of Na+ and decrease the concentration of K^+^ in serum, besides the concentration of copper ion (Cu^2+^) in plasma is decreased, while Zn^2+^ is increased during heat stress (14).

### Impacts on Antioxidant Capacity *in vivo*

**Figure 2:**
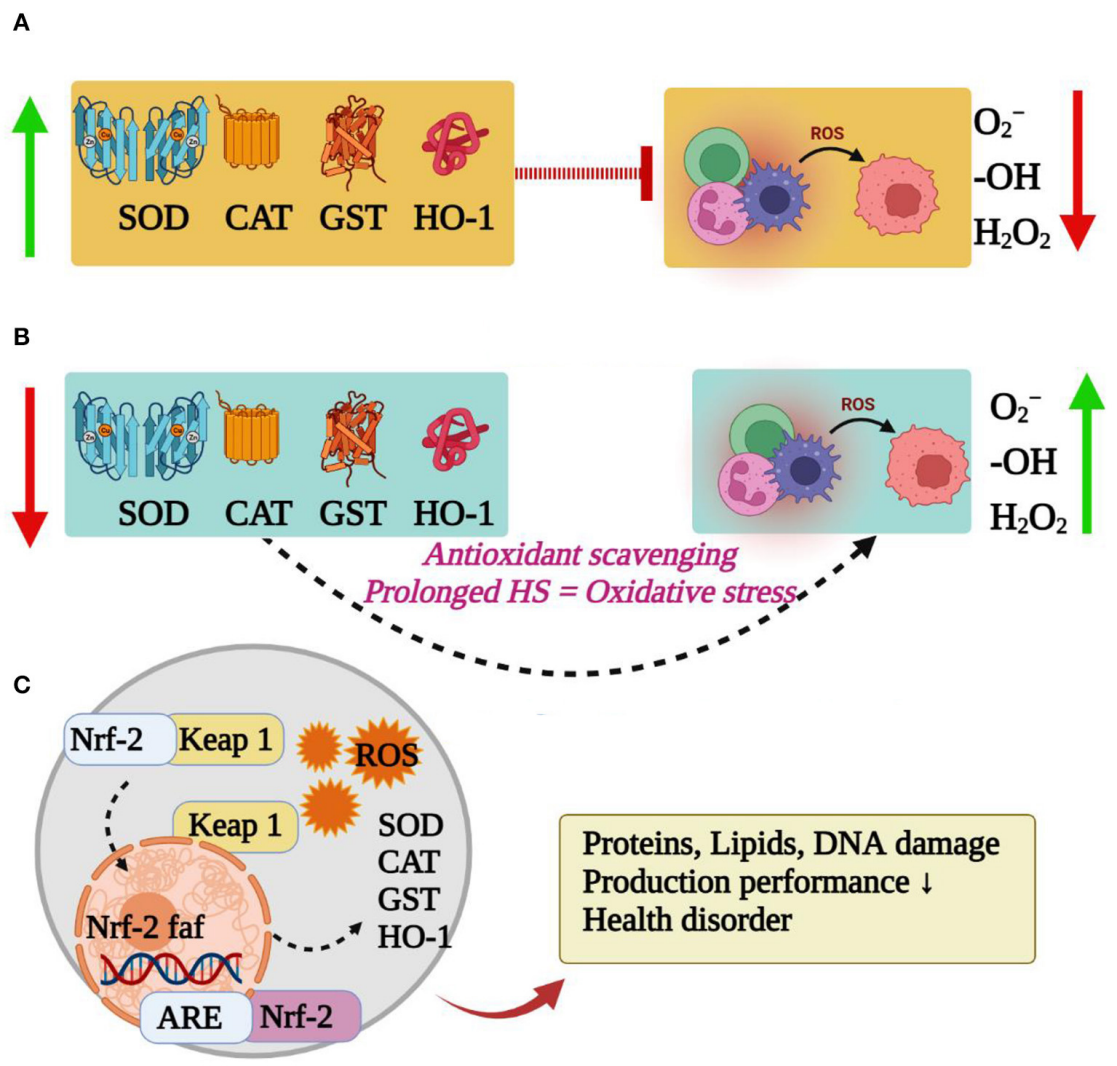
Effects of heat stress on antioxidant capacity and related signaling pathway of rabbits. **(A)** Thermoneutral temperature. **(B)** Heat stress. **(C)** Nrf pathway-Induction of oxidative stress.

The produced free radicals are removed to maintain a dynamic balance in the body during thermoneutral temperature. As summarized in Figure 2, the redox balance is broken by heat stress, and a large number of reactive oxygen species (ROS) and their metabolites (ROS-M) are released to the blood of rabbits, which are prone to oxidative stress (31). The nuclear factor erythroid 2-related factor 2 (Nrf2) is a key factor in cellular oxidative stress response and a central regulator of cellular antioxidants (32). The expression of Nrf2 is parallel to the degree of oxidative stress to maintain the balance of the redox state in the body (33). The increased levels of ROS by heat stress, and the downstream target genes of Nrf2, including catalase enzyme (CAT), superoxide dismutase (SOD), glutathione sulfurtransferase (GST) and heme oxygenase (HO-1), are expressed to against oxidative stress (34–38). These antioxidant enzymes, such as SOD and CAT, can remove substances with strong oxidative activity, such as oxygen ion (O^2−^) and hydrogen peroxide (H_2_O_2_), through the synergistic effect in the body to maintain the physiological function and prevent cell damage (39). However, the concentration and activity of SOD and CAT in rabbits are decreased when rabbits exposed to heat stress (30, 40, 41). For instance, the activities of SOD and CAT in the pituitary and hypothalamus of New Zealand rabbits were decreased along with the higher temperature and longer exposure time (42). Interestingly, the antioxidant enzymes activities in the body temporarily increase in the early stage of heat stress by accelerating the clearance of oxygen free radicals and reducing the production of lipid peroxidation products; however, long-term and/or high-intensity of heat stress causes the increase of oxygen free radicals, and an excessive load of the Nrf2 pathway leads to a decrease in antioxidase activity and induces oxidative stress (43), thereby inducing an excessive accumulation of free radicals in rabbits and damaging all the components of the cell, including proteins, lipids and DNA (39). Then the concentration of serum metabolites (Malondialdehyde, MDA) causing organ damage had also been changed by heat stress in rabbits (44). The oxidative stress in rabbits is associated with lower production performance, severe health disorders and biological damage (45, 46). Hence, reducing oxidative stress is key means for alleviating the heat stress in rabbits.

### Impacts on Reproductive Performance

The reproductive performance of rabbits is an important trait that affecting the economic benefits of rabbit production. The high reproductive performance leads to short generation interval in rabbit which provide the needed animal protein with low capital outlay and time (47). The most suitable temperature for rabbit reproduction ranges from 15 to 20°C. If the ambient temperature exceeds this range, rabbits are prone to stress and diseases, affecting their reproductive performance negatively (48).

#### Female Rabbits

The growth of embryos, pregnancy rate, litter size, litter weight and milk yield of female rabbits are affected by heat stress (49). The high temperature significantly reduces estrogen secretion and causes irregular estrus, it can cause abnormal morphology of egg cells, such as cytoplasmic shrinkage and rupture of the transparent membrane, making egg cells unable to fertilize and affecting the reproduction of female rabbits (20). In order to increase heat dissipation, pregnant rabbits flow a large amount of blood to the skin, the blood in the uterus and umbilical cord is greatly reduced during heat stress, which causes the serious insufficient blood supply to the fetus and embryo sac, and then the embryo becomes smaller, affecting the fetus growth and leading to a high mortality rate (48). The weight of pregnant rabbits and litter is significantly decreased during heat stress (49). Pregnant rabbits are sensitive to mutant heat stress, high temperatures above 35°C can cause salivation and neurological symptoms, and even abortion in pregnant rabbits (11). In addition, the lactation of female rabbits was adversely affected by high ambient temperature compared with the thermoneutral temperature during late pregnancy (24, 50).

#### Male Rabbits

Male rabbits are more sensitive to high temperatures than female rabbits. The synthesis and secretion of hypothalamic gonadotropin-releasing hormone (GnRH) are inhibited under high temperature, which significantly affects the function of testis and decreases the semen quality in male rabbits (51, 52). The ejaculation volume of young male rabbits decreased by 80%, the sperm vitality decreased by 75%, and the number of sperm per mL of semen decreased by 92% during heat stress due to the 51 days sperm production cycle of male rabbits and the 8–13 days sperm storage time in the epididymis (30). The temporary infertility of male rabbits lasts 45–70 days during heat stress, which is one of the reasons for the reproductive difficulties of male rabbits in autumn (53). Heat stress can affect the semen quality due to the accumulation of free radicals in the gonad of male rabbits, resulting in the damage of the antioxidant system (41). The semen quality decreases due to the heat stress that leads to a series of physiological and biochemical reactions in the testis, which change the internal microenvironment of the testis, the structural abnormalities of ROS, heat stress protein (HSP), mitochondrial, smooth endoplasmic reticulum etc. (52, 54). The integrity of DNA is destroyed, which induces the changes of sperm chromatin conformation and DNA methylation, thus damaging the spermatogenesis system and affecting the reproductive performance of male rabbits (54).

### Impacts on Production, Carcass and Meat Quality

**Figure 3:**
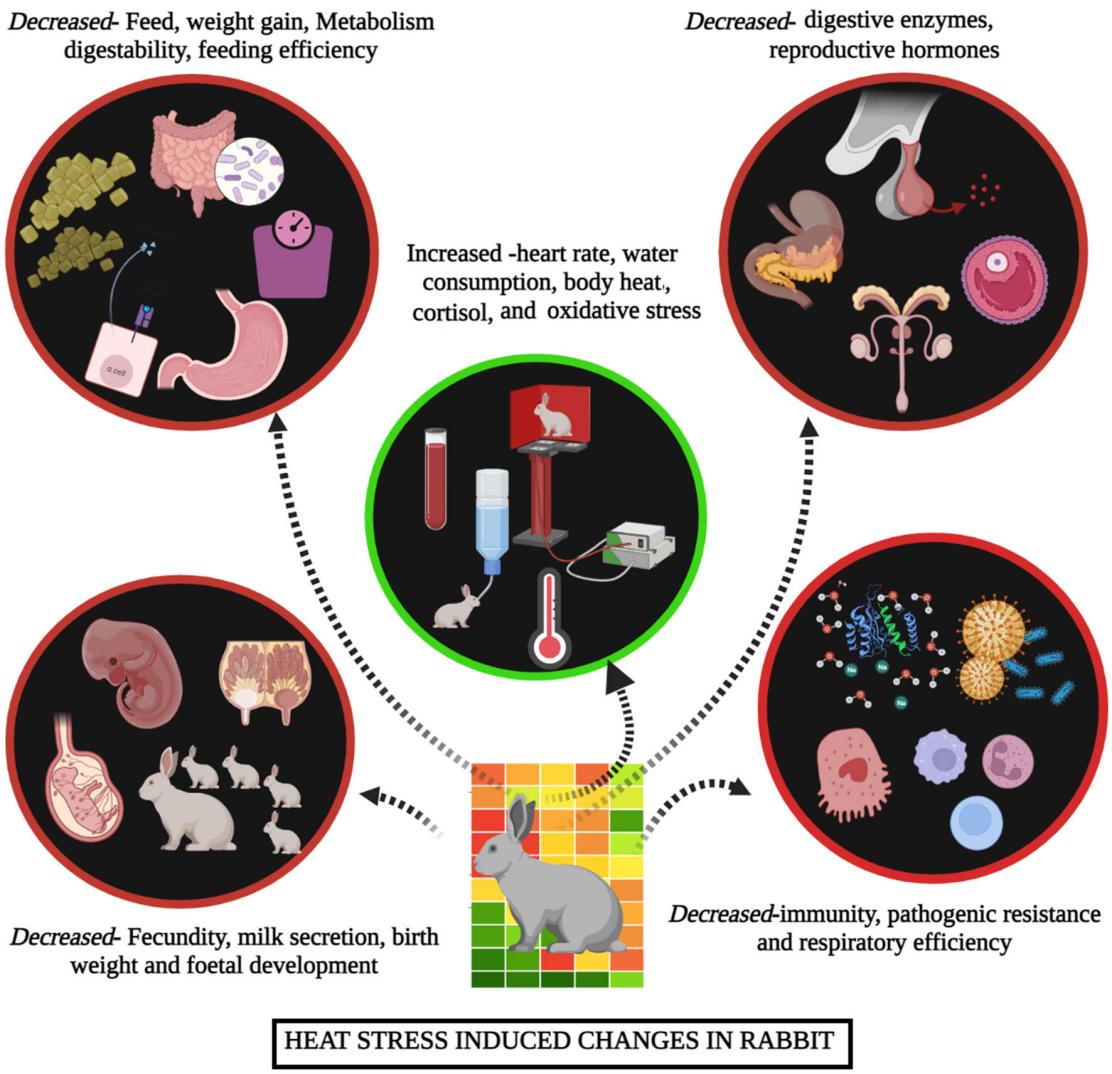
Summarize the impacts of heat stress on rabbits.

As summarized in Figure 3, when rabbits are exposed to heat stress, they dissipate excess heat produced inside the body manifested by specific behavioral and physiological changes in rabbits. Heat stress severely reduces the feed intake in rabbits due to the effect on the feeding center of the lower thalamus and heat increment (55). The sympathetic nerve resulting in reduced gastrointestinal function can decrease food intake during heat stress (21). Decreased feed intake results in a lower supply of nutrients, thus reducing the weight and growth rate in rabbits; besides, decreased endocrine and antioxidant capacity can lead to reduced immune function, decreased fat and protein deposits, organ and cell inflammation, growth rate and reproduction and increased mortality that can significantly decrease the heath, meat and hair production in rabbits (56–60).

Carcass and meat quality traits of rabbits, such as tenderness and color, are crucial to consumer acceptance (1). To strengthen the knowledge of how heat stress affects the carcass and meat quality traits is important to promote rabbit meat production. Heat stress affects the maintenance of heat balance in rabbits, resulting in the change of physiological adjustment and biochemical profile (61), which may decrease the production performance and affect the carcass and meat quality traits (62). Previous study has shown that the redness and yellowness of meat are decreased along with the increment of ambient temperature, which also increase the cooking loss and lead to the reduction of meat juiciness (63). Excessive ambient temperature depressed slaughter and carcass weight at the fixed market age, it can be explained that heat stress reduce the feed intake and performance of growing rabbits, and cause the dysplasia of gastrointestinal tract (64). On the contrary, the reference carcass percentage has a positive linear relationship with ambient temperature, which can be attributed to the decreased relative portion of metabolic active organs (chest, liver and kidney, etc.) along with the aggravation of heat stress (63). In summary, heat stress causes profound impact on production performance, carcass and meat quality of rabbits. Therefore, mitigation measures should be taken to minimize the negative impact of heat stress on rabbit production.

## Potential Mitigation Strategies of Nutritional Intervention

### Feeding Strategies to Prevent Heat Stress

Compared with morning and evening, the appetite of rabbits is decreased in the summer noon. Therefore, the feeding strategy of rabbits needs to be combined with its physiological status to adjust the feeding time and amount. The specific strategies are to appropriately increase the feeding in the morning and evening, less fed or feeding of the green and juicy feeds are used at noon (40, 65–67). The green and juicy feeds can alleviate heat stress in rabbits. Feeding dandelion, Ixeris sonchifolia, watermelon peel and fruit peel can decrease heat stress and relieve thirst and diuresis, adding an appropriate amount of welsh onion and garlic to the rabbit diet can prevent rabbit coccidiosis and enteritis (65). Evaporative heat dissipation by increasing the breathing rate is an important way to dissipate heat during heat stress for the rabbit, which can cause water loss *in vivo* (68). Studies have shown that an adequate supply of drinking water can alleviate heat stress in rabbits. Stephan reported that when the temperature increased from 18 to 38°C, and the water requirement of rabbits increased by 50% (69). The earlier study found that the water intake of female rabbits at 30°C was 10.7% higher than the temperature of the control group (20°C) (70). It is better to provide low-temperature drinking water and add an appropriate amount of salt for rabbits, which can make up for the consumption of electrolytes *in vivo* and reduce heat stress (71). According to Marai et al. (49), the water intake, respiratory rate and rectal temperature of rabbits were decreased, but the body weight, feed intake and feed conversion rate were increased in rabbits after drinking cold water at 10–15°C during heat stress. However, excessive drinking water is unnecessary after the evaporation reaches its maximum level. Drinking too much water leads to increased urination, washing away the nutrients in the digestive tract, resulting in a decrease in nutrient retention and hindering the growth of rabbits (11). Taken together, the possible feeding and nutritional strategies in relieving heat stress impacts on rabbits have been shown in Table 1.

**Table 1:** The beneficial effects of nutritional strategies in heat-stressed rabbit.

**Supplements**	**Dose**	**Beneficial effects on heat-stressed rabbits**	**References**
Green and juicy food	–	Decrease heat stress and relieve thirst and diuresis, prevent rabbit coccidiosis and enteritis	(65)
Ice water	–	The body weight ↑, feed intake and feed conversion rate ↑	(49)
Fat	3,050 Kcal DE kg^−1^ of diet	Production performance (e.g., body weight, daily weight gain, litter size and weight, reduce pre-weaning mortality)↑	(12, 72, 73)
	–	Improve the palatability of feed, the appetite ↑, feed intake and growth rate ↑	(74)
Amino acid	50–100 mg kg^−1^ of diet	Haemato-biochemical and reproductive indicators in male rabbits↑	(75)
**Vitamins**			
Vitamin C	500 mg kg^−1^ of diet	(1) T3, T4 ↑cortisol and glucose levels, body temperature↓	(76)
	200 mg kg^−1^ of diet	(2) Corticosterone and MDA in blood ↓	(77)
	200 mg kg^−1^ of diet	(3) Reverse the liver and kidney dysfunction	(78)
	200 mg kg^−1^ of BW	(4) mRNA and protein expression of HSP70 in liver and kidney tissues ↑ cortisol and leptin ↓, satiety signal ↑	(79)
	1g L^−1^ in DW	(5) Productive and reproductive performance, oocyte maturation rate↑	(80)
Vitamin E	40 mg kg^−1^ of diet	(1) Semen quality; T3, T4 hormonal levels↑, body temperature and respiration rate↓.	(70, 81)
	320–640 mg kg^−1^ of diet	(2) T-AOC ↑	(82)
	150 mg kg^−1^ of diet	(3) Semen quality, plasma glucose, high-density lipoprotein↑ plasma cholesterol and triglyceride ↓	(83)
	100 mg kg^−1^ of diet	(4) Pregnancy rate, litter size, lipid metabolism, antioxidant capacity ↑	(84)
Vitamin A	750 IU of diet	(1) Antioxidant enzyme activity ↑, lipid peroxidation ↓	(11, 15)
	1.2–1.5 times higher than NRC (1977)	(2) Skin temperature, rectal temperature and pulse ↓	(24)
**Electrolyte**			
NaHCO_3_	300 mg kg^−1^ of BW	(1) Dissolving mucus, invigorating the stomach, inhibiting acid and increasing appetite	(85)
	–	(2) Sodium↑ and respiratory alkali poisoning ↓	(86, 87)
	0.1–0.2% in DW	(3) Invigorate the stomach and regulate blood acid-base balance	(88)
KCl	–	(1) Maintains intracellular osmotic pressure and acid-base balance	(89)
	0.3–0.5% in DW	(2) Relieve heat stress, replenish potassium loss, and maintain blood potassium concentration.	(24)
KHCO_3_	–	(1) Maintain the blood potassium concentration and improve the HCO_3_- concentration	(87)
	2.5% in diet	(2) Production performance and blood biochemical indexes ↑	(11)
NH_4_Cl	2% in DW	Recover the high blood pH value, regulate acid-base balance and prevent respiratory alkali poisoning	(90, 91)
**Minerals**			
Zinc	1.5 times higher than NRC (1977)	(1) Anti-heat stress agent, trace elements in milk ↑, litter size and weight ↑	(92)
	1 mg subcutaneous injection	(2) The lipid metabolism function ↑	(93)
Chromium	0.8–1.0 mg kg^−1^ of diet	(1) Alleviate the damage on testicular spermatogenic function testosterone, LH and FSH in serum ↑	(94)
	0.4 mg kg^−1^ of diet	(2) Daily gain and feed intake ↑, cellular immunity function ↑	(95)
Selenium	0.3 mg kg^−1^ of diet	(1) Rectal temperature ↓, serum total protein content, albumin content and GSH-Px activity ↑. The total protein, albumin, ALT, fructose and total antioxidant capacity ↑, MDA ↓	(96)
	25–50 mg kg^−1^ of diet	(2) Body weight and average daily gain ↑, GSH and CAT activities ↑and reduce MDA ↑	(94)
Chinese herbal medicines	–	(1) Immunologic enhancement and antivirus action	(97)
	20 mg injection	(2) Lymphocyte proliferation and antibody titer ↑	(98)
	1–3% in diet	(3) Production performance, reproductive performance and antioxidant level ↑	(99, 100)
	1% in diet	(4) Mortality ↓	(101)
	5% in diet	(5) Intestinal mucosa structure ↑	(102)
	4.4 g in diet	(6) Sperm yield and survival rate ↑, sperm malformation rate ↓	(103)
**Functional active substances**			
L-Carnitine	50 mg kg^−1^ of diet	(1) Rectal temperature, heart rate, and respiration rate ↓, growth indices, feed conversion ratio, blood hemoglobin, white blood cell counts, total protein, glucose, and red blood cell counts ↑	(104)
	100 mg kg^−1^ of diet	(2) Sperm motility, serum antioxidative status ↑	(105)
Algae	100 mg kg^−1^ of diet	(1) Production performance, antioxidants indices ↑, inflammatory responses and intestinal pathogens ↓	(106)
	300 mg kg^−1^ of diet	(2) Conception rate, kindling rate, litter size, embryo quality and the ovulatory response ↑	(84)
Essential oils	300 mg kg^−1^ of diet	Plasma testosterone concentration and GSH activity, sperm output, sperm cell counts, intact acrosome, sperm normality ↑, sperm cells with abnormal tail and plasma MDA contents ↓	(107)
	100–150 mg kg^−1^ of diet	Haemato-biochemicals constitutes, immunologic status, antioxidant capacity, and fertility traits ↑, lipid peroxidation ↓	(108)
Plant extract	100–300 mg kg^−1^ of diet	(1) Growth performance, carcass traits and antioxidant status ↑	(109)
	5–10 g kg^−1^ of diet	(2) Body weight, feed intake, SOD, T-AOC and GSH-Px ↑, MDA and cortisol content ↓	(110)
	50 mg kg^−1^ of BW	(3) Heat tolerance, oxidative status and semen quality ↑	(111)
	1,000 mg kg^−1^ of diet	(4) Hematological and antioxidative indicators ↑	(112)
	10 mg kg^−1^ of BW	(5) Redox status, hormonal balance, total litter size, kindling rate, litter size at birth and litter weight at birth ↑	(113)
Tannins	10 g·kg^−1^ of diet	Body weight, feed intake, SOD, T-AOC and GSH-Px activities ↑, MDA and cortisol levels ↓	(114)
	5 or 10 g·kg^−1^ of diet	Growth performance, carcass and meat quality traits ↑, lipid peroxidation of meat ↓	(115)

*BW, body weight; DW, drinking water*.

*Arrow, ↑: increase, ↓: decrease*.

### Adjusting Feed Formula

The feed intake of rabbits is significantly decreased during heat stress, resulting in insufficient nutrients and energy intake (12). Thus, increasing the dietary energy levels can be used to alleviate the adverse effects of heat stress, it has been found that the energy level in the rabbit diet should be 0.95 MJ·kg^−1^ higher than the recommended value of National Research Council (NRC) (1977) during summer (70). The energy level in the diet is improved by adding fat in the rabbit industry usually (116). Previous studies indicated that using high energy level feed can significantly improve the production performance (e.g., body weight, daily weight gain, litter size and weight, reduce pre-weaning mortality) and the serum albumin in rabbit during heat stress (16, 72, 73). Supplementing fat to the diet could also improve the palatability of feed, thereby increasing the appetite of rabbits (74). Increased appetite could ameliorate the decreased feed intake by heat stress and enhance the growth rate of rabbits. In a recent study, high energy feed was used to improve the heat stress, results showed that the rectal temperature, heart rate, and respiration rate decreased significantly in rabbits during heat stress (104). Moreover, due to the high unsaturated fatty acid content in vegetable fat helps rabbits to against heat stress, adding 3% vegetable fat to the diet can reportedly relieve the rabbit from heat stress (12).

During protein digestion, heat is produced in the metabolic process due to the serious heat increment, and the heat dissipation is greatly decreased during heat stress (117). Therefore, feeding a low protein diet and balanced amino acid pattern could be used to alleviate the heat stress of rabbits (118). Abdelnour et al. (75) suggested that increased the proline levels in feed could improve the haemato-biochemical and reproductive indicators in male rabbits during heat stress. The phycocyanin (100 mg/kg) which rich in amino acids was used to enhance the performance, antioxidants indices, decrease inflammatory responses and intestinal pathogens of growing rabbits during heat stress (106). Besides, it has been demonstrated that using plant protein in diet is better than that of animal protein under heat stress. When rabbits are subjected to heat stress, plant proteins could be selected according to the amino acid standard to prepare diets for reducing the adverse effects of heat stress (119).

In addition to adjusting the energy and protein concentrations in the feed formula, it has been found that the pellet feed has high nutrient density and ideal palatability, which can also improve the production performance of heat-stressed rabbits (11). Under heat stress situation, there was weak digestive capacity for rabbits, supplementing green forage in the feed formula can increase the cellulose and vitamins in digestive tract, improving appetite and digestion of rabbits (12). Therefore, an appropriate feed formula and form are beneficial for heat-exposed rabbits and relieving the detrimental effects of heat stress.

### Application of Feed Additives

#### Vitamins

Vitamin C, known as ascorbic acid, provides anti-stress and antioxidant function (106). It has been wildly known that vitamin C supplementation can mitigate heat stress and increase the growth performance in rabbits (120). Vitamin C participates in the redox reaction and eliminates the free radicals produced by peroxidation *in vivo* to prevent tissue cells from oxidative damage (121). Vitamin C promotes the synthesis of antibodies, enhances the phagocytosis of leukocytes, and improves the detoxification ability of the liver (122). Studies have shown that dietary inclusion of vitamin C inhibits the increased body temperature in heat-stressed rabbits, increases the T3 and T4 levels, and decreases the concentration of cortisol and glucose, corticosterone and MDA in blood, thereby reducing the detrimental impact of heat stress *in vivo* (76, 77, 123). Based on previous report, adding 150–200 mg·kg-1 vitamin C to the diet improved the production performance of rabbits and reversed the liver and kidney dysfunction caused by heat stress (78). It has also been reported that vitamin C improves the mRNA and protein expression of heat shock protein (HSP70) in liver and kidney tissues, and reduces cortisol and leptin, improving the satiety signal in heat-stressed rabbits (79). On the other hand, adding fresh tap water supplemented daily with vitamin C (1 g/L) improves the productive and reproductive performance, and also the oocyte maturation rate (66–80%) in rabbit exposed to heat stress conditions (80).

Vitamin E is an intracellular antioxidant, and its lipid solubility makes it a suitable membrane antioxidant against oxidative damage, maintaining the function of the cell membrane system and reducing the release of creatine kinase in muscle cells during stress, thereby preventing excessive calcium influx and interfering with normal cell metabolism (124). Vitamin E can alleviate the immunosuppression caused by the release of adrenal cortex hormone at high temperatures and promote the synthesis of immunoglobulin to improve the body's disease resistance and reduce mortality (125), vitamin E can also reduce the serum cortisol levels, thereby reducing rabbit's adverse reactions to heat stress (126). Studies showed that adding vitamin E to the water increased the normal sperm, volume, count, motility, live sperm and T3, T4 hormonal levels, then decreased the rectal, skin, ear temperature and respiration rate, thus improving the fecundity of male rabbits (81). However, vitamin E supplementation had no significant effect on reducing the body temperature of rabbits under heat stress (24). Zhang et al. (82) found that vitamin E improved the total antioxidant capacity (T-AOC) of rabbits under acute heat stress. Similarly, Hashem et al. (83) indicated that inclusion of 150 mg·kg^−1^ vitamin E alleviated the negative impact of heat stress on semen quality, and increased the plasma glucose and high-density lipoprotein concentrations of male rabbits, but the plasma cholesterol and triglyceride concentration have been decreased during heat stress. It has been suggested that 100 mg·kg^−1^ vitamin E supplementation in female rabbits' diet could improve the pregnancy rate and litter size of heat-stressed rabbits, and increase the lipid metabolism and antioxidant capacity (84). However, feeding high doses of vitamin E causes the lack of other fat-soluble vitamins (117).

Vitamin A is associated with innate immunity, T cell proliferation and antibody production (127, 128). According to previous studies, vitamin A improves the body's antioxidant enzyme activity, reduces the degree of lipid peroxidation caused by free radicals, and enhances the rabbit's resistance during heat stress (11, 15). It has been found that feeding vitamin A to 3-month-old rabbits with 750 IU could decrease the skin temperature, rectal temperature and pulse of heat-stressed rabbits (24). Suggesting that the use of vitamin A could improve the growth performance and health of rabbits under heat stress. In addition, vitamin D, vitamin K and nicotinic acid also play an important role in regulating heat stress response and preventing high body temperature (129, 130), but more researches about these vitamins are needed.

#### Electrolyte

Because of the dense hair and absence of sweat glands, rabbits use their ears and respite to decrease the body temperature during heat stress (6). The dramatically increased respiration rate in response to heat stress causes a serious acid-base imbalance in rabbits by discharging excess CO_2_ (85, 117). Therefore, the appropriate addition of sodium bicarbonate (NaHCO_3_), potassium chloride (KCl), potassium bicarbonate (KHCO_3_) and ammonium chloride (NH_4_Cl) to the diet or drinking water of rabbits plays an important role in restoring the acid-base balance of rabbits under heat stress. The NaHCO_3_ is a kind of electrolyte additive and acid-base regulator, which equips the function of dissolving mucus, invigorating the stomach, inhibiting acid and increasing appetite (131, 132). It can be used to supply sodium and can work as the main buffer material in blood and tissue to decrease respiratory alkali poisoning and improve the ability to resist heat stress (86, 87). According to Zhou (88), NaHCO_3_ can be used to invigorate the stomach and regulate blood acid-base balance, adding 0.1–0.2% NaHCO_3_ to drinking water could reduce the loss of heat stress. KCl maintains intracellular osmotic pressure and acid-base balance (89). The secretion of the adrenocorticotropic hormone is increased in rabbits, thereby promoting the excretion of potassium by glomerular, resulting in decreased blood potassium levels (133, 134). Previous study has shown that supplementation of 0.3–0.5% KCl to drinking water could relieve heat stress, replenish potassium loss, and maintain blood potassium concentration (24). The KHCO_3_ can be used to maintain the blood potassium concentration and alleviate the HCO_3_- concentration caused by heat stress (87). It had been observed that dietary inclusion of KHCO_3_ (2.5%) improved the production performance and blood biochemical indexes of heat-stressed rabbits (11). NH_4_Cl was also reported to recover the high blood pH value caused by heat stress, which regulates acid-base balance and prevents respiratory alkalosis in rabbits (90, 91).

#### Zinc

The activity of more than 300 enzymes (e.g., oxidoreductases, transferases, hydrolases, lyases, isomerases, and ligases) requires zinc as substrates (135). It is also involved in the metabolism of enzymes in the body (136, 137). Zinc is related to the antioxidant defense system, immune function and skeletal development, a low level of zinc increases the oxidative damage of membrane caused by free radicals (138). Zinc supplementation inhibits free radicals because it is a part of SOD, GSH, GST and HO-1 (139). Moreover, zinc plays an essential role in the synthesis of metallothionein, which acts as a free radical scavenger (140, 141). Accordingly, zinc is an important element that needs to be added to feed when heat stress occurs in livestock and poultry (142). Zinc as an anti-heat stress agent mainly exists in the form of zinc monocarbonate (ZnCO_3_), zinc sulfate monohydrate (ZnSO_4_·H_2_O), zinc pyridine acid (ZnPic), bacitracin zinc, granular coated bacitracin zinc, amino acid zinc, and so on. They can act alone or interact with each other and are combined with vitamins for anti-heat stress. It is noted that the use of bacitracin zinc should be permitted by the local law. The release of trace elements to milk was increased when zinc was upregulated by 50% in the rabbit diet under heat stress, and the litter size and weight were significantly affected by Zn addition (92). Because of high temperatures, the lipid metabolism of animals is disordered, resulting in fat deposition; dietary supplementation of zinc could improve the lipid metabolism function in rabbits under heat stress, this may be related to the fact that zinc is involved in the synthesis of lipid metabolism enzymes (93).

#### Chromium

Chromium is an essential mineral, which is an integral component of chromodulin and also necessary for insulin functioning (143). The chromium is able to promote glucose transporter 4 (GLUT4) to transfer from cytoplasm to the cell membrane *via* activating insulin receptors, which in turn facilitates the entry of glucose into cells (144). Besides, chromium is an important component of the glucose tolerance factor (GTF), which affects the metabolism of glucose, lipids, proteins and nucleic acids by enhancing insulin activity (145). Supplementing 0.8–1.0 mg·kg^−1^ yeast chromium to the rabbits' diet alleviated the negative effects of heat stress on testicular spermatogenic function, luteinizing hormone (LH) and follicle stimulating hormone (FSH) in the serum of male rabbits (94). Huang et al. (95) demonstrated that organic chromium could significantly increase daily gain and feed intake of heat-stressed rabbits, and supplementation of 0.4 mg·kg^−1^ organic chromium to feed could improve the production performance, and inclusion of 1.6 mg·kg^−1^ organic chromium could be used to improve the cellular immunity function.

#### Selenium

Selenium is involved in many key physiological processes, such as reproduction, immunity and growth, and is an essential trace element for mammals (146). Selenium is an important component of at least 25 different selenium proteins, such as glutathione peroxidase (GSH-Px) and thioredoxin reductase (TrxRs) (147, 148), and the GSH-Px is a major phase II detoxification enzyme and can reduce the lipid peroxide level. Therefore, an appropriate amount of selenium in rabbit diets is crucial for the anti-oxidation and immune function of rabbits (149). Previous report suggested that inclusion of 0.034 mg·kg^−1^ organic selenium to the diet of heat-stressed rabbits decreases their rectal temperature by about 0.5°C. On the contrary, the serum total protein content, albumin content and GSH-Px activity were increased. Also, the total protein, albumin, alanine aminotransferase (ALT), fructose content and total antioxidant capacity in seminal plasma were increased, while the MDA content was significantly decreased (96). Adding biosynthesized nano-selenium (25 or 50 mg·kg^−1^) to the feed could increase the body weight and average daily gain of the rabbit under heat stress, improve GSH and CAT activities and reduce the serum MDA content (150). Considering the higher biological activity of nano selenium, future researches should focus more on the relieving effect of nano-selenium on heat-stressed rabbits.

#### Compound Chinese Herbal Medicines

Chinese herbal medicines and their ingredients equip immunologic enhancement and antivirus action, which can be used as immunopotentiator or anti-infection drugs (97). Chinese compound herbs have been suggested to promote lymphocyte proliferation and enhance antibody titer in rabbits (98). Numerous studies have indicated that the addition of some heat-clearing, detoxicating, bactericidal and disease-resistant Chinese herbal medicines, such as *Radix Bupleuri, Rhizoma Coptidis* and *Artemisia annua*, to the diet of rabbits can reduce the problems associated with heat stress and improve the production performance, reproductive performance and antioxidant level of heat-stressed rabbits (99, 100). The traditional Chinese medicine formula (including *Radix Rehmanniae, Rhizoma Coptidis*, mulberry bark, etc.) was reported to decrease the rabbit's mortality during heat stress (101). Wang et al. (102) found that using 5% Chinese herbal compounds (e.g., Huoxiang, *Atractylodes, Rhizoma Coptidis*, etc.) to the feed of New Zealand rabbits reduced the damage of rabbit intestinal mucosa structure during heat stress. In addition, Li et al. (103) have found that Wuzi Yanzong Pill could increase the reproductive capacity of male rabbits in summer, thus improving the sperm yield and survival rate, and reducing the sperm malformation rate. Chinese herbal medicines contain active ingredients, such as flavonoids, polysaccharides, polyphenols, alkaloids, etc., which exhibits antioxidant, anti-inflammatory, antibacterial and antiviral properties (97). This could explain the role of Chinese herbal medicines in relieving heat stress in rabbits. However, due to the composition of Chinese herbal medicines is complex, which may lead to inconsistent conclusions in the application. Further researches on the use of Chinese herbal medicines to ameliorate heat stress in rabbits are still required; in particular, there is a need to elucidate the active ingredients in Chinese herbal medicines.

#### Functional Active Substances

At present, the functional active substances, including L-carnitine, algae, essential oils, plant extract, tannins etc., which have been got growing attentions and were also used to improve the health and production in rabbit during heat stress.

L-carnitine (LC) is a functional additive which work as an important role in fatty acid metabolism and energy production (151). It plays an essential role in the oxidation of mitochondrial fatty acids by transporting long-chain fatty acids like acylcarnitine esters through the inner mitochondrial membrane (152). Supplementation of 50 mg of LC kg^−1^ in basal diet could decrease the rectal temperature, heart rate, and respiration rate, whereas enhance the growth rate and feed conversion ratio in heat-stressed rabbit; rabbit fed LC contained diet also showed higher blood hemoglobin, white blood cell counts, total protein, glucose and red blood cell counts, compared with those fed basal diet (104). Adding 100 mg·kg^−1^ LC to male rabbit diet was observed to improve heat stress-induced impairment of semen quality (105). These beneficial effects may be related to LC improving the antioxidant capacity and up-regulating HSP expression (153).

Algae have been exhausted for several years as nourishment for people and animals owing to the outstanding nutritious profile and great carotenoid substance (154). The algae like spirulina platensis usually employed as a food complement and a good source of vitamins and proteins (155). It has been reported that the phycocyanin (the main active components of spirulina platensis) ameliorates lipid peroxidation and inhibits the generation of pro-inflammatory cytokines (IL-1, IL-6, and TNF-a) and the activities of inducible nitric oxide synthase (iNOS) as well as cyclooxygenase 2 (COX-2) enzymes (156). Phycocyanin (100 mg·kg^−1^ in basal diet) was also reported to enhance the growth performance, antioxidants indices and decrease the inflammatory responses and intestinal pathogens in heat-stressed rabbit (106). A recent study showed that Spirulina platensis (300 mg·kg^−1^ in basal diet) increases the conception rate, kindling rate, litter size, embryo quality and the ovulatory response (corpora lutea number and ovulation rate) in rabbit during heat stress (84). Therefore, the mode of action of algae in alleviating heat stress impacts of rabbits was possibly due to its anti-inflammatory and antioxidant activity (155).

The essential oils have been widely used as effective feed additives for promoting the growth performance in animals, and they appear many beneficial aspects such antimicrobial and antioxidant functions (156). Adding 0.5–1.5 mL·kg^−1^ grape seed oil to basal diet can increase the body weight and feed intake in rabbit (157). El-Ratel et al. (107) demonstrated that extra virgin olive oil supplementation (300 mg·kg^−1^) increases plasma testosterone concentration and GSH activity, sperm output, sperm cell counts, intact acrosome, sperm normality, while reduces sperm cells with abnormal tail and plasma MDA contents in male rabbits under heat stress. Abdelnour et al. (108) found that inclusion of 100–150 mg·kg^−1^ thyme essential oil in the diet improves the haemato-biochemicals constitutes, immunologic status, antioxidant capacity, reduces the lipid peroxidation, thus enhancing the fertility traits (e.g., litter size, viability rate at birth, viability rate at weaning) and milk production. The positive effects of essential oils mainly attributed to the antioxidant activity (156). However, certain types of essential oils have a characteristic odor that may affect the rabbit's appetite, which should be avoided in application.

Plant extract has also been used to decrease the heat stress in recent year. Grape seed extract contains a lot of polyphenols, which are powerful free radical scavengers and antioxidants to decrease the oxidative stress and DNA damage (158). It has been revealed that the grape seed extract against heat stress to maintain performance, carcass traits and antioxidant status in rabbit (109). Authors attributed their findings to the presence of procyanidin from the grape seed extracts, which reduced heat stress impacts and improved rabbit's health (159). Procyanidin is a type of flavonoids and have been reported that can trap lipid peroxides and free radicals, chalate to free iron molecules (inhibit iron-induced lipid peroxidation), non-competitively inhibit xanthine oxidase (a major generator of free radicals) (110). These characteristic of procyanidin can be used to interpret how the grape seed extract improve rabbit's health from heat stress. Furthermore, El-Desoky et al. (111) reported that *Moringa oleifera* leaves ethanolic extract (MLEE) supplementation at a level of 50 mg·kg^−1^ body weight could be effectively used to enhance heat tolerance, oxidative status and semen quality of rabbit bucks under heat stress. Al-Sagheer et al. (112) suggested that addition of 1,000 mg·kg^−1^ in diet could improve the hematological and antioxidative indicators, and efficiently mitigate the detrimental effects of chronic temperature stress on performance, hematobiochemical features, and oxidative stability. El-Desoky et al. (113) indicated that nanoencapsulated MLEE has 30 active components, which alleviates (supplemental dose of 10 mg·kg^−1^ body weight) the negative impacts of heat stress by improving metabolism, redox status, and hormonal balance of rabbit does during summer, thereby increase the reproductive performance (e.g., total litter size, kindling rate, litter size at birth and litter weight at birth). The mode of action of plant extract is complex due to it contains a variety of active components, which still needs further in-depth researches.

Tannins are a group of polyphenolic compounds, which consist of aromatic rings with one or more hydroxyl groups, which can combine with free radicals to form resonance-stabilized phenoxyl radicals, this structure indicate the strong antioxidant properties of tannins (160). It has been reported that the body weight, feed intake, SOD, T-AOC and GSH-Px activities were increased, and the MDA and cortisol levels were decreased by tannins supplementation (10 g·kg^−1^ of diet) in heat-stressed rabbit (114). Liu et al. (115) suggested that addition of 5 or 10 g·kg^−1^ of tannins in diet could improve the growth performance, carcass and meat quality traits, and certain stress parameters in rabbits reared under high ambient temperature, and the tannins also showed inhibitory effects on heat stress-induced lipid peroxidation of rabbit meat.

The role of functional active substances in relieving heat stress of rabbits is due to the biological activities, such as antioxidant, anti-inflammatory, antibacterial functions, therefore, the bioactive substances have broad application prospects in rabbits under heat stress situation. However, existing reports mainly researched the protective effects of active substances on growth rate, carcass traits, meat quality, reproductive performance, redox status, and immune response of heat-stressed rabbits, the study of molecular mechanism is lacking. In future studies, it is necessary to elucidate the underlying mechanisms of the beneficial effects of active substances in rabbits under heat stress. Meanwhile, it is also worthwhile to study the mitigation effect of active substances on heat stress-induced impairment of intestinal barrier function in rabbits.

## Conclusions

In summary, heat stress has been a severe challenge for modern rabbit industry, especially in the tropical and subtropical regions. Heat stress results from several factors (e.g., high environmental temperature and humidity, high stocking density), which causes a series of unfavorable changes in immune function, endocrine, blood biochemical indexes and antioxidant capacity, thus negatively affecting the production performance (e.g., growth rate, carcass and meat quality, reproductive performance) in rabbits. Therefore, researchers need to further study the physiological change caused by neuroendocrine under heat stress and figure out a holistic approach to attenuate the detrimental effect of heat stress on rabbits. The potential use of feeding strategies and nutritional regulation could be beneficial to ameliorate heat stress. Further studies should be attempted on the combination of several approaches for relieving heat stress and evaluating the efficiency and economic benefit in rabbit production.

## Author Contributions

BB and W-CL: conceptualization and writing—review and editing. Z-LL, SP, and FC: selection and collection of bibliographies, and the data curation. Z-LL and FC: writing—original draft preparation. W-CL: supervision, project administration, and funding acquisition. All authors have read and agreed to the published version of the manuscript.

## Conflict of Interest

The authors declare that the research was conducted in the absence of any commercial or financial relationships that could be construed as a potential conflict of interest.

## Publisher's Note

All claims expressed in this article are solely those of the authors and do not necessarily represent those of their affiliated organizations, or those of the publisher, the editors and the reviewers. Any product that may be evaluated in this article, or claim that may be made by its manufacturer, is not guaranteed or endorsed by the publisher.
